# Physical Examinations of Employees

**Published:** 1914-05

**Authors:** 


Physical Examinations of Employees.—The Department of of Health is compiling a list of corporations and firms which employ physicians for the purpose of making periodical physical examinations of employees. Our readers will confer a favor upon the department by sending the name of any corporation or firm which should be included in this list, adding, wherever possible, the name of the physician in charge of the work, and the date of its inauguration. Communications on this subject should be addressed to Dr. S. S. Goldwater, Commissioner of Health, and should be marked Personal.—*New York Medical Journal.*

